# Cultural Differences in Diet and Determinants of Diet Quality in Switzerland: Results from the National Nutrition Survey *menuCH*

**DOI:** 10.3390/nu11010126

**Published:** 2019-01-09

**Authors:** Giulia Pestoni, Jean-Philippe Krieger, Janice Marie Sych, David Faeh, Sabine Rohrmann

**Affiliations:** 1 Division of Chronic Disease Epidemiology, Epidemiology, Biostatistics and Prevention Institute, University of Zurich, Hirschengraben 84, CH-8001 Zurich, Switzerland; giulia.pestoni@uzh.ch (G.P.); jean-philippe.krieger2@uzh.ch (J.-P.K.); david.faeh@uzh.ch (D.F.); 2 Institute of Food and Beverage Innovation, ZHAW Zurich University of Applied Sciences, Einsiedlerstrasse 34, CH-8820 Wädenswil, Switzerland; sych@zhaw.ch; 3 Health Department, Bern University of Applied Sciences, Falkenplatz 24, CH-3012 Bern, Switzerland

**Keywords:** dietary patterns, diet quality scores, 24h dietary recall, language regions

## Abstract

Sociodemographic differences in dietary consumption were observed in different populations. The current study aimed to identify sociodemographic and lifestyle determinants of diet quality and to investigate the differences in diet quality between the three main language regions of Switzerland. Using data of the Swiss National Nutrition Survey *menuCH* (*n* = 2057), two diet quality scores—Alternate Healthy Eating Index and Mediterranean Diet Score—were computed. Linear regression models were used to investigate the determinants of diet quality and chi-square tests were used to test for differences in single score components between language regions. Significantly higher diet quality scores were observed for individuals who were female, older, normal weight, non-Swiss, with tertiary education or moderate-to-high physical activity level. Additionally, residents of the French- and Italian-speaking parts of Switzerland scored higher than residents of the German-speaking region. More specifically, the higher diet quality observed in the French- and Italian-speaking regions was mediated by higher scores in the components of alcohol, dairy products, fat, fish, sugar-sweetened beverages and whole grains. The present results may help to better characterize population groups requiring specific dietary recommendations, enabling public health authorities to develop targeted interventions.

## 1. Introduction

Diet is widely considered as one of the most important factors influencing health-related outcomes. In fact, several studies reported unbalanced nutrition to be a major risk factor for the development of chronic diseases such as cardiovascular diseases, cancers and diabetes [1,2,3]. Traditional approaches in nutritional epidemiology focused primarily on the intake of single dietary components. However, as food is not eaten in isolation, these approaches failed to consider the complexity of the diet and the possible synergistic or antagonistic effect of different nutrients [4,5,6].

Dietary pattern analysis has recently become the preferred strategy in nutritional analysis, believed to better represent overall diet. A common approach to investigate overall diet quality is the use of a priori dietary patterns, i.e., diet quality scores based on existing nutrition knowledge and dietary recommendations [4,6,7]. Two widely used diet quality scores are the Alternate Heathy Eating Index (AHEI), which aims to assess the adherence to the Dietary Guidelines for Americans, and the Mediterranean Diet Score (MDS), which investigates the adherence to the traditional Mediterranean diet [8,9]. A high diet quality, as assessed by both the AHEI and the MDS, was associated with a lower risk for morbidity and mortality of major chronic diseases [3,4,10,11]. Although the two diet quality scores are highly similar with respect to some components, they include a unique dietary combination and are computed differently. The MDS is calculated using sex-specific medians as cut-offs and is, therefore, highly dependent on the population under study. This ensures a homogenous contribution of different components to the overall score, but does not allow for comparisons across different populations or the investigation of changes over time [12]. In contrast, the AHEI is based on fixed cut-offs, and therefore allows for such comparisons [12]. For this reason, both diet quality scores were investigated in the present study.

A strong influence of various sociodemographic and lifestyle factors (such as age, sex, education, body mass index (BMI) and physical activity level) on diet quality was observed in different populations [13,14,15]. These sociodemographic and lifestyle determinants of dietary consumption are, therefore, important to identify at the national level, since they can contribute to characterize vulnerable population groups and help public health authorities to develop targeted nutritional campaigns [16]. Cultural differences also play an important role among the sociodemographic and lifestyle factors influencing diet quality. In fact, several studies observed a substantial diversity in dietary patterns across European countries [17,18,19]. However, comparisons of nutritional data are often limited by differences in data collection and processing, or by differences in national health policies and health care systems [20,21,22]. Switzerland represents a unique setting to overcome these issues. In fact, Switzerland consists of three main language regions (German-speaking, French-speaking and Italian-speaking regions), with corresponding cultures partly influenced by the respective neighboring countries, but with common national policies and health care system [20,21,22]. Since chronic diseases are known to be strongly influenced by the individuals’ dietary habits, in depth investigation of diet quality across Swiss language regions could help to explain regional differences in the distribution of chronic disease morbidity and mortality observed in previous Swiss studies [20,21,22,23].

In Switzerland, the effects of sociodemographic and lifestyle factors on diet quality have been investigated in different studies [20,24,25,26,27,28,29,30]. However, since no nutrition survey existed in Switzerland until 2015, most of these results relied on undetailed dietary data or were derived from a single city or region [20,24,25,26,28,29,30]. To fill this gap, the first Swiss National Nutrition Survey *menuCH* was recently conducted with the aim to assess the dietary habits in a representative sample of the Swiss population [27,31]. These newly available data allowed us to overcome the aforementioned limitations.

The aims of the current study were, therefore, to identify sociodemographic and lifestyle determinants of diet quality in Switzerland and to investigate the differences in diet quality between the three main language regions.

## 2. Materials and Methods

The “Strengthening the Reporting of Observational Studies in Epidemiology—Nutritional Epidemiology (STROBE-nut)” checklist was used to report the findings of the present study [32].

### 2.1. Study Design and Setting

The Swiss National Nutrition Survey *menuCH* is a cross-sectional population-based survey, conducted in 10 study centers across Switzerland between January 2014 and February 2015. A detailed description of the recruitment procedure and participation rate was published elsewhere [27,31]. Briefly, Swiss residents aged 18 to 75 years were drawn from a stratified random sample provided by the Federal Statistical Office, intended to be representative for the following 35 strata (7 × 5): the seven administrative regions of Switzerland (Lake Geneva, Midlands, Northwest, Zurich, Eastern, Central and Southern Switzerland), covering the three main language regions (German-, French- and Italian-speaking region) and five age groups (18–29, 30–39, 40–49, 50–64 and 65–75 years old) [27].

From a gross sample of 13,606 individuals, 5496 were successfully contacted by mail or phone and 2086 agreed to participate in the study (38% net participation rate). Among the 2086 participants, 2057 had a complete dietary assessment (i.e., two 24-h dietary recalls (24HDR)) and were finally included in the study. A complete flow diagram of study participation was previously published [27].

The survey protocol was approved by the ethics committee of the canton of Lausanne (Protocol 26/13) and by the corresponding regional ethics committees. All procedures performed in the survey followed the ethical standards of the Declaration of Helsinki and all participants provided a written informed consent. The survey was registered at the ISRCTN registry under the number 16778734 [33].

### 2.2. Dietary Assessment

The dietary assessment of *menuCH* consisted of two non-consecutive 24HDR, conducted by trained dietitians [31]. The first 24HDR was administered face-to-face, whereas the second one was conducted by phone two to six weeks later. Overall, the interviews were distributed across all seasons and weekdays [27]. Food consumption of the *menuCH* participants was recorded in a standardized and automated way using the software GloboDiet^®^ (formerly EPIC-Soft^®^, version CH-2016.4.10, International Agency for Research on Cancer (IARC), Lyon, France) [34,35], adapted to Switzerland (GloboDiet^®^ trilingual databases dated 12.12.2016, IARC, Lyon, France; Federal Food Safety and Veterinary Office, Bern, Switzerland). To facilitate quantification of food consumption, a photo book illustrating portion sizes and common household measures was used during the 24HDR [36]. Detailed information about dietary supplement use was not recorded. For quality control purposes, the dietitians’ compliance to several survey-specific standard operating guidelines was assessed. Additionally, GloboDiet^®^ data were cleaned and screened for inconsistencies applying the recommendations of IARC [27]. Despite quality control, some under- and over-reporting persisted in the *menuCH* data. Chatelan et al. [27] observed indeed 16.9% of plausible under-reporting and 1.5% of plausible over-reporting among the 2057 participants. However, since the 24HDR could also represent a special occasion, in which the participants had particularly low or high food consumption, and to be able to apply the weighting strategy to the data, all 2057 participants were included in the analyses.

After data collection, foods, recipes and ingredients recorded with GloboDiet^®^ were linked to the most appropriate item in the Swiss Food Composition Database [37], using the matching tool FoodCASE (Premotec GmbH, Winterthur, Switzerland), allowing for a direct estimation of macronutrient and micronutrient intake by the study participants.

### 2.3. Diet Quality Scores

Diet quality of the *menuCH* participants was assessed using two diet quality scores: the 2010 version of the AHEI and the 2003 version of the MDS [8,9].

Originally created in 2002, the AHEI was based on the Healthy Eating Index, which aims to assess the adherence to the Dietary Guidelines for Americans. The updated 2010 version of the AHEI was further based on discussions with nutrition experts and on a comprehensive literature review, which allowed us to identify foods and nutrients associated with lower risks of major chronic diseases [8]. The AHEI includes 11 components: vegetables, fruits, whole grains (defined as a carbohydrates-to-fiber ratio ≤ 10:1), sugar-sweetened beverages and fruit juices, nuts and legumes, red and processed meat, trans fat, fish (as a proxy for long-chain n-3 fatty acids), polyunsaturated fatty acids (PUFA), sodium and alcohol. The score of single components can range from 0 to 10 points, with intermediate food intakes scored proportionately between the minimum and the maximum score. Overall, the total AHEI can range from 0 to 110 points, with 0 meaning minimal adherence and 110 meaning maximal adherence [5,8].

The MDS was constructed in 1995 with the aim of assessing adherence to the traditional Mediterranean diet and was revised in 2003 to include fish intake [9]. The score includes nine components: vegetables, legumes, fruits and nuts, cereals, fish, meat, dairy products, fat intake (ratio of monounsaturated to saturated fatty acid) and alcohol. For each component, a value of either 0 or 1 point is assigned to the participants depending on different cut-offs. For all components except alcohol, sex-specific medians were used as cut-offs. For the component alcohol, intakes between 10 to 50 g per day for men and between 5 to 25 g per day for women are considered as optimal. Overall, total MDS can range from 0 to 9 points, with 0 meaning minimal adherence and 9 meaning maximal adherence to the traditional Mediterranean diet [5,9].

In the present study, both AHEI and MDS were computed separately for each of the two 24HDR and the mean of the two interviews was used for the analyses. A detailed description of the food groups included and the scoring system for each diet quality score is presented in Tables S1 and S2.

### 2.4. Sociodemographic and Lifestyle Variables

Different sociodemographic and lifestyle variables were considered as possible determinants of the participants’ dietary patterns.

Information about sociodemographic and lifestyle variables was obtained from a questionnaire filled out by the participants at home and checked for completeness and clarity by the dietitians on the day of the first 24HDR. Age was calculated from the self-reported birth date and the date of the first interview, and divided into four categories (18–29, 30–44, 45–59, 60–75 years). The language region was determined by the participants’ canton of residence (German-speaking region: Aargau, Basel-Land, Basel-Stadt, Bern, Lucerne, St. Gallen, Zurich; French-speaking region: Geneva, Jura, Neuchatel, Vaud; Italian-speaking region: Ticino). The physical activity level was assessed using the short version of the International Physical Activity Questionnaire (IPAQ) and divided into three categories (low, moderate, high). Moreover, the following sociodemographic and lifestyle variables were further considered as potential determinants of diet quality: sex; nationality (Swiss, Swiss binational, non-Swiss); education (primary or no degree, secondary, tertiary); household type (living alone, couple without children, couple with children, one-parent family with children, adult living with parents, others); gross household income (<6000, 6000–13,000, >13,000 Swiss francs/month); smoking status (never, former, current); self-reported health (very bad to medium, good to very good); currently on a weight loss diet (yes, no).

Body weight and height of the participants were measured according to international standard protocols [27,38] and used to calculate the BMI. For study participants who were pregnant (*n* = 14) or lactating (*n* = 13), or when measurements were impossible (*n* = 7), self-reported weight (weight before pregnancy for pregnant and lactating women) and/or self-reported height were used to estimate BMI. In the present analysis, the participants were categorized according to the World Health Organization guidelines: underweight (BMI < 18.5 kg/m^2^), normal weight (18.5 kg/m^2^ ≤ BMI < 25.0 kg/m^2^), overweight (25.0 kg/m^2^ ≤ BMI < 30.0 kg/m^2^) and obese (BMI ≥ 30.0 kg/m^2^).

### 2.5. Weighting Strategy

In order to take into account the sampling design and non-response, and to extrapolate the results of the survey to the entire Swiss population, weighting factors were applied to the data according to the *menuCH* weighting strategy [39]. In the current study, all results were weighted for sex, age, marital status, major area of Switzerland, nationality and household size. Additionally, analyses including food consumption were further weighted for season (spring, summer, autumn, winter, according to the mean date between the two 24HDR) and weekday (two 24HDR during weekdays [Monday to Thursday], two 24HDR during weekend days [Friday to Sunday], or one 24HDR during weekdays and one during weekend days). After weighting, the 2057 participants included in the study were representative of a total population of 4,627,878 individuals.

### 2.6. Statistical Analysis

For the calculation of some AHEI and MDS components, data about the micronutrient consumption of the *menuCH* participants were needed. However, the Swiss Food Composition Database used to assess the nutrient composition of the consumed food items contains incomplete micronutrient information. To address this problem, single food items were assigned to the food categories defined in the GloboDiet^®^ software. The missing micronutrient values of the single food items were then imputed using the median micronutrient value of the given food categories. A sensitivity analysis was performed to compare AHEI and MDS scores obtained with the median imputation approach to those obtained when replacing missing micronutrient values by 0.

For the data analysis, descriptive statistics were first used to characterize the survey’s participants overall and by language region. To investigate the agreement between the AHEI and the MDS, a Spearman partial correlation coefficient was calculated, considering the average energy intake of the participants. Two linear regression models were then used to investigate the determinants of diet quality, as assessed by the two a priori dietary patterns. In order to increase the number of participants with complete information about sociodemographic and lifestyle factors, multivariate imputation by chained equations (*m* = 25) was performed [40]. Finally, the differences in dietary patterns across the three main language regions of Switzerland were investigated in greater depth. In order to perform the same analysis for both diet quality scores, the components of the AHEI were first divided into tertiles. Since the components were often not normally distributed, a subdivision in equal groups was not always possible. For skewed distributions, individuals with the same amount of points were allocated in the same group, and the other groups were formed using the tertile cut-offs or creating two groups of similar size. Chi-square tests were then used to investigate the overall differences and the differences between language regions for each component of AHEI (categorized into low, medium and high tertile) and MDS (categorized into 0, 0.5 and 1 points). Bonferroni correction was applied to adjust for multiple testing. Since the subdivision of a continuous variable in quantiles could potentially lead to inaccurate results, a sensitivity analysis was conducted, where the differences between language regions were investigated considering the AHEI components as continuous and tested using Kruskal–Wallis tests.

The analyses were conducted using R software (version 3.4.1. for Windows, R Foundation for Statistical Computing, Vienna, Austria). Multivariate imputation by chained equations was performed with the package *mice* [40]. Additionally, the package *survey* was used to perform the weighted statistical analyses [41]. For all analyses, the statistical significance was set at *p* < 0.05.

## 3. Results

### 3.1. Characteristics of the Participants

The characteristics of the *menuCH* participants overall and by language region are presented in Table 1. The majority of the individuals was normal weight (54.1%), Swiss (61.4%) and had secondary or tertiary education (42.6% and 52.6%, respectively). Moreover, most participants never smoked (42.9%), had a good to very good health status (87.1%) and did not follow a weight loss diet (94.4%). A higher percentage of participants with a non-Swiss nationality was observed in the French-speaking region compared to the overall population. Additionally, individuals living in the Italian-speaking part of Switzerland had a lower education level, a lower gross household income and reported a lower health status.

### 3.2. Determinants of Diet Quality

Considering the energy intake of the participants, a moderate positive correlation was observed between the two scores (Spearman ρ = 0.55, *p*-value < 0.01).

Several sociodemographic and lifestyle factors were statistically significant determinants of diet quality, with high level of agreement between the two diet quality scores (Table 2). In general, female sex, older age, non-Swiss nationality, tertiary education, moderate-to-high physical activity level and being on a weight loss diet were associated with higher diet quality. By contrast, being overweight and obesity were associated with lower diet quality.

**Figure 1:**
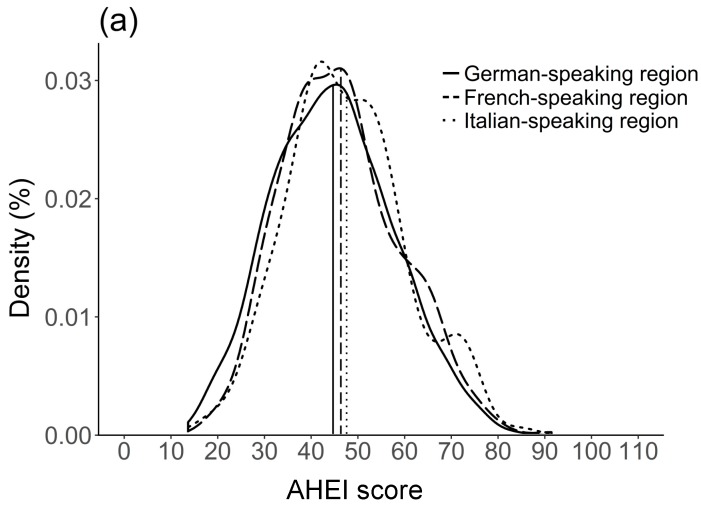

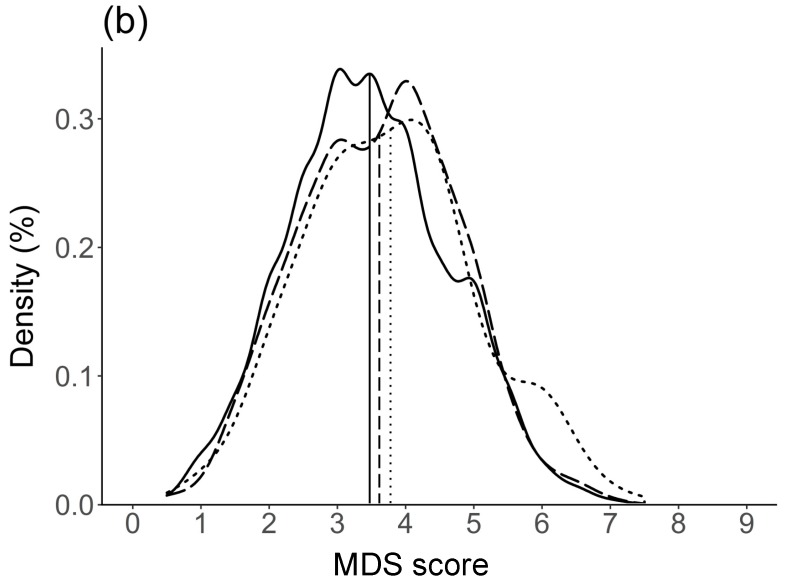
Weighted density plots of (**a**) Alternate Healthy Eating Index (AHEI) and (**b**) Mediterranean Diet Score (MDS) by language region (*n* = 2057). The density plot is a smoothed representation of a histogram and shows the distribution of a variable. In the graph, the total area under the curve is 1 (i.e., the integral of the variables is scaled to 1). The density plot allows for a direct comparison of the language regions, although each region has a different number of participants. The density plots were weighted for sex, age, marital status, major area of Switzerland, nationality, household size, season and weekday. Vertical lines represent the weighted mean for each language region.

Furthermore, residents of the French- and Italian-speaking regions of Switzerland had a higher diet quality compared to residents of the German-speaking region. The distribution of the two diet quality scores by language region is represented in Figure 1. The weighted means of the AHEI were 44.7 points (standard deviation [SD] = 12.8), 46.4 points (SD = 12.5) and 47.6 points (SD = 12.6) for German-, French- and Italian-speaking regions, respectively. Likewise, weighted means of the MDS were 3.5 points (SD = 1.2), 3.6 points (SD = 1.1) and 3.8 points (SD = 1.3), respectively. The overall weighted mean was 45.3 points (SD = 12.8) for the AHEI and 3.5 points (SD = 1.2) for the MDS.

### 3.3. Differences in Single Components of Diet Quality Scores by Language Region

As in the previous analyses, high level of agreement was observed between the dietary components of AHEI and MDS. Table 3 shows the results for single components of the AHEI. Overall, statistically significant differences between language regions were observed in the components whole grains, sugar-sweetened beverages/fruit juices, trans fat, fish, sodium and alcohol. 

The results for single components of the MDS are shown in Table 4. Overall, statistically significant differences were found in the components fish, dairy products, alcohol and fat intake.

### 3.4. Sensitivity Analyses

In the first sensitivity analysis, AHEI and MDS were computed replacing the missing micronutrient values by 0. This analysis revealed an overall weighted mean of 44.3 points (SD = 12.7) for the AHEI and of 3.3 points (SD = 1.2) for the MDS, which are very similar to the weighted mean obtained when using the median imputation approach.

In the second sensitivity analysis, the differences across language regions for the AHEI were investigated considering the single components as continuous and tested using Kruskal–Wallis tests (Table S3). Overall, the analysis revealed very similar significant results to those obtained when dividing the AHEI components in tertiles. The only differences in significance were observed for the components sodium (German vs. French: *p*-value = 0.01 when considering the AHEI as continuous and *p*-value = 0.11 when dividing the AHEI components into tertiles) and alcohol (German vs. Italian: *p*-value = 0.01 when considering the AHEI as continuous and *p*-value = 0.08 when dividing the AHEI components into tertiles).

## 4. Discussion

In the current study, several sociodemographic and lifestyle factors were important determinants of diet quality as assessed by AHEI and MDS. Notably, statistically significant differences were observed across language regions, with participants living in the French- and Italian-speaking regions scoring higher than participants living in the German-speaking region. Due to its multicultural setting, Switzerland represents an optimal scenario to investigate cultural habits. For this reason, the differences in diet quality across language regions were investigated in greater depth by examining single components of the two diet quality scores. The analysis revealed significant differences across language regions for the components whole grain, sugar-sweetened beverages, fat, fish, alcohol and dairy products.

Interestingly, there was a certain overlap in the determinants of diet quality as assessed by AHEI or MDS. The two scores showed, indeed, a moderate level of agreement. As mentioned before, although some components of AHEI and MDS are highly similar, the two diet quality scores are computed differently. The AHEI is based on fixed cut-offs and, therefore, allows for comparisons across different populations [12]. To our knowledge, there are no comparable AHEI data computed for the populations of the neighboring countries Germany, France and Italy. However, the mean AHEI score obtained in the present study (45.3 points [SD = 12.8]) was similar to the score observed in a US, UK and Chinese population [42,43,44]. A lower mean AHEI score was instead observed for a subgroup of the Swiss population in a previous study (32.9 points [SD = 10.1] for women and 30.6 points [SD = 9.9] for men) [24]. Nevertheless, the authors used the original version of the AHEI, which differs to some extent from the 2010 version used in the present study, and the component trans fat was not included in the score [24]. For these reasons, the results are not directly comparable.

In the current study, 11 out of 12 potential determinants of diet quality were found to be significantly associated with one or both diet quality scores. As already reported from many previous studies [13,15,24,25,28,42,45], a higher diet quality was observed for females and older participants. The weak significant association between sex and MDS in the present study was most likely due to the use of sex-specific medians in the calculation of the score. Confirming earlier reports in Switzerland [24,25,26,30], the current results indicated higher scores among non-Swiss participants, which could depend on their country of origin. In fact, a high proportion of non-Swiss *menuCH* participants originates from Italy, France, Portugal, Spain or other southern European countries (37.2%), where the traditional Mediterranean diet is typically consumed. Moreover, in the present study, significantly higher diet quality scores were observed among individuals with a high educational level, a finding in line with previous literature [13,24,25,26,28,42,46]. This could be explained by a high level of food and nutrition literacy in highly educated individuals [13,14,47]. However, in contrast to what is observed in many studies [13,14,42], the current analysis did not show any association between household income and AHEI or MDS. Finally, different behavioral risk factors were often found to be interrelated and cluster with each other [28,48], a concept supported by the results of the present study. In fact, in our analysis, a high level of physical activity and following a weight loss diet were associated with higher diet quality, whereas overweight and obesity as well as a very bad to medium self-reported health were associated with lower diet quality. Additionally, an association between diet quality and smoking status was found. Current smokers had a lower AHEI score compared to never smokers. On the contrary, former smokers had a higher MDS compared to never smokers, which might be related to an overall increased health-consciousness after quitting smoking [48,49].

Interestingly, in the present study, significant associations were observed between language regions and diet quality scores. Although very similar regression coefficients were observed in the French- and Italian-speaking regions (2.03 vs. 1.99, respectively), the association between the Italian-speaking region and AHEI was not statistically significant. However, given the relatively low number of *menuCH* participants living in the Italian-speaking region, the power to identify significant differences was probably insufficient.

The differences in diet quality between language regions might be related to the cultural habits typical of the large neighboring countries of Switzerland. The analyses investigating the differences in diet across language regions for the single components of the diet quality scores indicate that a high proportion of individuals living in the German-speaking region had a high score for the component whole grains, but a low score for the components sugar-sweetened beverages/fruit juices, fat, fish and dairy products. Similar results were observed in a previous *menuCH* analysis [27], in which the consumption of different food groups (instead of dietary patterns) was investigated. Moreover, similar patterns were observed in the European Prospective Investigation into Cancer and Nutrition (EPIC) study [17], where the German centers had a higher consumption of butter, margarine and juices, as well as a lower consumption of vegetable oils and fish products compared to other European centers. In contrast, residents of the French- and Italian-speaking regions in our analysis had more favorable dietary patterns, with characteristics typical of the traditional Mediterranean diet. Similar results were again observed in the previously mentioned studies [17,27]. In fact, low consumption of milk and trans fat, moderate fish consumption and high alcohol consumption were also observed among Swiss-French and French [17,27]. Additionally, a low consumption of soft drinks, juices, butter, margarine and dairy products as well as a moderate alcohol consumption were observed among Swiss-Italians and in the Italian EPIC centers [17,27].

In addition to diet quality scores, another common approach used in nutritional epidemiology for investigating overall diet quality is the identification of a posteriori or data-driven dietary patterns. These patterns are indeed directly derived from dietary data, usually by principal component or cluster analyses [12]. The complementarity of these methods and the importance of using various approaches to investigate the overall diet quality is often emphasized in the literature [10,12,50]. In fact, both a priori and a posteriori dietary patterns have strengths and limitations [12]. For this reason, a posteriori dietary patterns were used to assess the diet quality of the *menuCH* participants in a parallel study [51]. Sociodemographic and lifestyle determinants of four identified dietary patterns were investigated, showing similar results to those observed in the present study [51].

To our knowledge, this study is the first investigating a priori dietary patterns in a representative sample of the Swiss population. The participants were drawn from a national stratified random sample and a weighting strategy was applied to the data to correct for non-response and uneven distribution of the interviews across seasons and weekdays. The food consumption of the *menuCH* participants was assessed by two non-consecutive 24HDR using the reference software GloboDiet^®^, which was proven to provide reliable estimates of the consumed nutrients and foods [34,35]. Finally, since diet is one of the most important factors influencing health outcomes, the results of the present study might help to explain the differences in morbidity and mortality of major chronic diseases previously reported in various Swiss studies [20,22,23,52].

However, the present study also has some limitations. For organizational and financial reasons, the *menuCH* survey only included the 12 most populated Swiss cantons [27,31]. Despite similar characteristics observed between respondents and non-respondents, study participants were probably more health-conscious than non-participants, potentially leading to participation bias. Additionally, recall bias and under- or over-reporting in the 24HDR cannot be excluded [27]. The use of two 24HDR to assess habitual food consumption is considered to be valid at the population level, but might be inaccurate to assess the habitual diet at the individual level. The two 24HDR in the present study may, therefore, have led to under- or overestimation of the diet quality scores. However, this most probably led to a non-differential information bias. Diet quality scores also have strengths and limitations. Although they are based on scientific evidence and current dietary recommendations, they only include selected components of the diet and are, therefore, only partially able to consider interactions between different dietary constituents [12,53]. Moreover, the components of diet quality scores are often equally weighted, assuming equal importance and therefore an additive impact on health [53,54]. Finally, although a median imputation approach was applied to the data due to missing micronutrient values, an over- or underestimation of the diet quality scores cannot be excluded.

## 5. Conclusions

In conclusion, data from the first *menuCH* survey allowed us to define major determinants of diet quality in a representative sample of the Swiss population. Notably, significant differences were observed across language regions, with participants living in the French- and Italian-speaking regions scoring higher than those living in the German-speaking region. More specifically, significant differences across language regions were mediated by the components whole grains, sugar-sweetened beverages, fat, fish, alcohol and dairy products. This underlines the potential influence of cultural habits typical of the large neighboring countries in a population with common national policies and health care system. Finally, these new results might help to explain regional differences observed in the morbidity and mortality of chronic diseases and to better characterize population groups that require specific dietary recommendations, enabling Swiss public health authorities to develop targeted interventions, such as reducing the sugar content of sugar-sweetened beverages or introducing taxes for alcoholic beverages in regions with high consumption.

## Figures and Tables

**Table 1:** Characteristics of the *menuCH* participants overall and by language regions ^a,b^.

	Overall	German-Speaking Region ^e^	French-Speaking Region ^e^	Italian-Speaking Region ^e^
**Number of participants**	2057	1341	502	214
**Weighted**	4,627,878	3,203,780	1,167,173	256,925
**Sex (%)**				
Males	49.8	51.1	46.5	48.4
Females	50.2	48.9	53.5	51.6
**Age groups ^c^ (%)**				
18–29 years	18.8	18.4	19.6	19.2
30–44 years	29.9	29.5	31.0	29.4
45–59 years	29.8	28.9	32.5	29.5
60–75 years	21.6	23.2	16.9	22.0
**BMI categories ^d^ (%)**				
Underweight	2.4	2.6	2.1	2.1
Normal weight	54.1	53.8	55.3	52.7
Overweight	30.6	30.8	30.2	29.9
Obese	12.9	12.8	12.4	15.4
**Nationality (%)**				
Swiss only	61.4	66.3	49.9	53.3
Swiss binational	13.8	12.4	18.0	11.9
Non-Swiss	24.8	21.3	32.1	34.7
**Education, highest degree (%)**				
Primary/no degree	4.7	3.6	6.4	10.3
Secondary	42.6	41.3	44.5	49.6
Tertiary	52.6	54.9	49.0	40.1
**Household type (%)**				
Living alone	18.1	16.3	22.7	20.0
Couple without children	31.7	34.4	24.9	29.0
Couple with children	32.8	31.4	36.1	35.1
One-parent family with children	4.4	3.9	6.0	3.2
Adult living with parents	7.1	6.7	7.9	8.9
Others	5.7	7.1	2.3	3.8
**Gross household income (%)**				
<6000 (CHF/month)	17.7	17.5	17.6	20.4
6000–13,000 (CHF/month)	39.8	41.1	37.3	35.8
>13,000 (CHF/month)	14.9	16.3	13.2	4.2
Did not answer	27.6	25.1	31.9	39.6
**Self-reported physical activity (%)**				
Low	12.9	13.9	9.7	14.6
Moderate	22.7	22.5	22.9	24.0
High	40.3	40.3	41.4	34.8
Did not answer	24.2	23.3	26.0	26.5
**Smoking status (%)**				
Never	42.9	43.3	42.3	40.4
Former	33.6	32.6	36.2	34.4
Current	23.3	23.8	21.3	25.2
**Self-reported health (%)**				
Very bad to medium	12.7	10.9	14.0	29.0
Good to very good	87.1	88.8	85.9	71.0
**Currently on a weight loss diet (%)**				
Yes	5.4	5.7	4.3	6.6
No	94.4	94.0	95.6	93.4

^a^ All results were weighted for sex, age, marital status, major area of Switzerland, nationality and household size. ^b^ Percentage of individuals with missing values < 0.5% are not shown. ^c^ Age groups are based on self-reported age on the day the sociodemographic and lifestyle questionnaire was filled. ^d^ Body mass index (BMI) was obtained from measured weight and height; self-reported weight or height were used when measurements were impossible; for pregnant and lactating women, self-reported weight before pregnancy was used. ^e^ German-speaking region: canton Aargau, Basel-Land, Basel-Stadt, Bern, Lucerne, St. Gallen, Zurich; French-speaking region: canton Geneva, Jura, Neuchatel, Vaud; Italian-speaking region: canton Ticino. CHF: Swiss francs.

**Table 2:** Association between diet quality scores and sociodemographic and lifestyle factors (*n* = 2057) ^a,b,c^.

	AHEI (0–110 Points)		MDS (0–9 Points)	
	Coefficient	95% CI	Coefficient	95% CI
**Sex**				
Males	0		0	
Females	**1.57**	**[0.38; 2.76]**	**−0.12**	**[−0.23; 0.00]**
**Age group ^d^**				
18–29 years	−1.31	[−3.07; 0.46]	−0.09	[−0.26; 0.08]
30–44 years	0		0	
45–59 years	**3.00**	**[1.64; 4.35]**	**0.22**	**[0.08; 0.35]**
60–75 years	**4.81**	**[3.13; 6.49]**	**0.34**	**[0.17; 0.50]**
**Language region ^e^**				
German-speaking	0		0	
French-speaking	**2.03**	**[0.82; 3.24]**	**0.20**	**[0.09; 0.32]**
Italian-speaking	1.99	[−0.27; 4.24]	**0.31**	**[0.09; 0.53]**
**BMI categories ^f^**				
Underweight	2.01	[−1.33; 5.35]	**0.36**	**[0.03; 0.68]**
Normal weight	0		0	
Overweight	**−2.56**	**[−3.77; −1.35]**	**−0.27**	**[−0.39; −0.15]**
Obese	**−4.60**	**[−6.28; −2.92]**	**−0.33**	**[−0.49; −0.17]**
**Nationality**				
Swiss only	0		0	
Swiss binational	1.06	[−0.47; 2.59]	0.09	[−0.06; 0.24]
Non-Swiss	**2.08**	**[0.78; 3.37]**	**0.22**	**[0.09; 0.34]**
**Education, highest degree**				
Primary/no degree	−1.23	[−3.75; 1.28]	−0.08	[−0.33; 0.16]
Secondary	0		0	
Tertiary	**2.47**	**[1.35; 3.59]**	**0.16**	**[0.05; 0.27]**
**Household type**				
Living alone	0.32	[−1.34; 1.99]	0.08	[−0.08; 0.24]
Couple without children	0		0	
Couple with children	−0.67	[−2.04; 0.69]	0.08	[−0.05; 0.21]
One-parent family with children	**−2.89**	**[−5.57; −0.20]**	−0.01	[−0.27; 0.25]
Adult living with parents	**−2.44**	**[−4.83; −0.05]**	−0.13	[−0.36; 0.11]
Others	**−3.11**	**[−5.56; −0.65]**	0.09	[−0.15; 0.33]
**Gross household income**				
<6000 (CHF/month)	−0.58	[−2.26; 1.09]	−0.10	[−0.25; 0.06]
6000–13,000 (CHF/month)	0		0	
>13,000 (CHF/month)	−0.26	[−1.88; 1.36]	−0.03	[−0.18; 0.13]
**Self-reported physical activity**				
Low	0		0	
Moderate	**2.45**	**[0.53; 4.37]**	**0.27**	**[0.09; 0.45]**
High	1.60	[−0.09; 3.29]	**0.22**	**[0.06; 0.38]**
**Smoking status**				
Never	0		0	
Former	−1.15	[−2.33; 0.03]	**0.12**	**[0.00; 0.23]**
Current	**−3.38**	**[−4.72; −2.04]**	−0.04	[−0.17; 0.09]
**Self-reported health**				
Good to very good	0		0	
Very bad to medium	**−1.89**	**[−3.49; −0.29]**	−0.05	[−0.20; 0.11]
**Currently on a weight loss diet**				
No	0		0	
Yes	**4.64**	**[2.35; 6.92]**	**0.29**	**[0.06; 0.51]**

^a^ Coefficients and 95% CI were derived from linear regression models; bolded values represent statistically significant results (*p*-value < 0.05); multiple imputation by chained equations was used to deal with missing values. ^b^ Coefficients equal to 0 represent the reference category. ^c^ All results were mutually adjusted for all the variables presented in this table, for mean energy intake, season and weekday, and weighted for sex, age, marital status, major area of Switzerland, nationality and household size. ^d^ Age groups are based on self-reported age on the day the sociodemographic and lifestyle questionnaire was filled. ^e^ German-speaking region: canton Aargau, Basel-Land, Basel-Stadt, Bern, Lucerne, St. Gallen, Zurich; French-speaking region: canton Geneva, Jura, Neuchatel, Vaud; Italian-speaking region: canton Ticino. ^f^ BMI was obtained from measured weight and height; self-reported weight or height were used when measurements were impossible; for pregnant and lactating women, self-reported weight before pregnancy was used. AHEI: Alternate Healthy Eating Index; CI: confidence interval; MDS: Mediterranean Diet Score.

**Table 3:** Weighted proportions of participants in the highest tertile of each Alternate Healthy Eating Index (AHEI) component and differences between language regions (*n* = 2057) ^a,b^.

	German-Speaking Region ^e^	French-Speaking Region ^e^	Italian-Speaking Region ^e^	Overall	German vs. French	French vs. Italian	German vs. Italian
	T3 (%)	T3 (%)	T3 (%)	*p*-Value ^c^	*p*-Value ^c,d^	*p*-Value ^c,d^	*p*-Value ^c,d^
Vegetables	32.8	34.1	36.7	0.49	1	0.44	0.36
Fruit	33.1	31.6	32.3	0.72	1	1	1
Whole grains	23.8	19.7	16.3	**<0.01**	0.50	**0.03**	**<0.01**
SSB/fruit juices ^f^	31.3	34.9	51.0	**<0.01**	1	**0.01**	**<0.01**
Nuts and legumes	34.1	31.4	32.5	0.07	0.56	0.71	0.05
Red/processed meat ^f^	32.2	35.7	35.7	0.39	1	1	0.75
Trans fat ^f^	83.2	91.8	95.0	**<0.01**	**<0.01**	0.97	**<0.01**
Fish	4.9	6.6	9.7	**<0.01**	**<0.01**	1	**0.01**
PUFA	33.5	34.1	27.0	0.18	0.55	0.50	0.86
Sodium ^f^	30.9	38.4	40.5	**0.03**	0.11	1	0.16
Alcohol ^f^	34.7	27.0	42.8	**<0.01**	**0.02**	**<0.01**	0.08

^a^ AHEI components were divided into tertiles; the proportion of participants in the highest tertile (i.e., best fulfilling the criteria for recommended intake) is represented for each language region. ^b^ All results were weighted for sex, age, marital status, major area of Switzerland, nationality, household size, season and weekday. ^c^ *p*-values were derived from chi-square tests; bolded values represent statistically significant results (*p*-value < 0.05). ^d^ Bonferroni correction was applied to adjust for multiple testing. ^e^ German-speaking region: canton Aargau, Basel-Land, Basel-Stadt, Bern, Lucerne, St. Gallen, Zurich; French-speaking regions: canton Geneva, Jura, Neuchatel, Vaud; Italian-speaking regions: canton Ticino. ^f^ For the components sugar-sweetened beverages/fruit juices, red/processed meat, trans fat and sodium, a high score corresponds to low consumption; for the component alcohol, a high score corresponds to moderate consumption. PUFA: polyunsaturated fatty acids; SSB: sugar-sweetened beverages; T3: third tertile.

**Table 4:** Weighted proportions of participants with 1 point in each Mediterranean Diet Score (MDS) component and differences between language regions (*n* = 2057) ^a,b^.

	German-Speaking Region ^e^	French-Speaking Region ^e^	Italian-Speaking Region ^e^	Overall	German vs. French	French vs. Italian	German vs. Italian
	1 Point (%)	1 Point (%)	1 Point (%)	*p*-Value ^c^	*p*-Value ^c,d^	*p*-Value ^c,d^	*p*-Value ^c,d^
Vegetables	27.6	30.0	30.1	0.60	1	1	1
Legumes	1.1	0.5	1.5	0.76	1	1	1
Fruits and nuts	32.8	32.9	29.6	0.42	1	0.25	0.50
Cereals	32.4	28.8	24.6	0.31	1	1	0.44
Fish	5.1	6.6	9.7	**<0.01**	**<0.01**	1	**0.01**
Meat ^f^	30.2	26.6	27.0	0.50	1	1	1
Dairy products ^f^	30.0	43.0	40.4	**<0.01**	**<0.01**	1	**0.03**
Alcohol ^f^	12.3	10.5	20.8	**0.05**	0.98	**0.01**	0.05
Fat intake ^g^	28.0	31.4	44.2	**<0.01**	1	**0.01**	**<0.01**

^a^ The proportion of participants with 1 point (i.e., best fulfilling the criteria for recommended intake) is represented for each language region. ^b^ All results were weighted for sex, age, marital status, major area of Switzerland, nationality, household size, season and weekday. ^c^ *p*-values were derived from chi-square tests; bolded values represent statistically significant results (*p*-value < 0.05). ^d^ Bonferroni correction was applied to adjust for multiple testing. ^e^ German-speaking region: canton Aargau, Basel-Land, Basel-Stadt, Bern, Lucerne, St. Gallen, Zurich; French-speaking regions: canton Geneva, Jura, Neuchatel, Vaud; Italian-speaking regions: canton Ticino. ^f^ For the components meat and dairy products, a high score corresponds to low consumption; for the component alcohol, a high score corresponds to moderate consumption. ^g^ Ratio of monounsaturated to saturated fatty acid.

## Supplementary Materials

The following are available online at http://www.mdpi.com/2072-6643/11/1/126/s1, Table S1: Components of the Alternate Healthy Eating Index, Table S2: Components of the Mediterranean Diet Score, Table S3: Weighted median (IQR) of each Alternate Healthy Eating Index (AHEI) component and differences between language regions (*n* = 2057).
