# Supplementary material for: Cultural Differences in Diet and Determinants of Diet Quality in Switzerland: Results from the National Nutrition Survey menuCH
Source: Nutrients. 2019 Jan 9;11(1):126. doi: 10.3390/nu11010126 (PMC6357532; doi:10.3390/nu11010126)
Supplement: Supplementary file 1 [file nutrients-11-00126-s001.pdf]

Table S1: Components of the Alternate Healthy Eating Index[a,b,c]

Components	Included food items	Criteria for minimum score (0)	Criteria for maximum score (10)	Overall Median [IQR]	German-speaking region Median [IQR]	French-speaking region Median [IQR]	Italian-speaking region Median [IQR]
Vegetables (servings/day)	All vegetables, leafy vegetables, sprouts, green beans, peas, sweat corn, root vegetables, cabbages, mushrooms, onions, seaweeds, legumes, soups; Except: olives, herbs, vegetables juices	0	≥ 5	3.0 [1.6 - 4.6]	3.0 [1.6 – 4.5]	3.1 [1.7 – 4.8]	3.0 [1.5 – 5.2]
Fruit (servings/day)	All fruits; Except: fruit juices, fruit jams, candied fruit	0	≥ 4	2.8 [0.9 – 5.1]	2.7 [0.9 – 5.2]	3.0 [1.0 – 5.0]	2.8 [0.2 – 4.7]
Whole grains (g/day)	All bread products, flours, cereal flakes and bran, dough, pasta, rice, spätzle, other cereal grains (quinoa, barley, ...) with a carbohydrates-to-fibre ratio ≤ 10:1			2.7 [0.0 – 5.0]	3.0 [0.0 – 5.0]	2.5 [0.0 – 5.0]	0.0 [0.0 – 5.0]
Women		0	≥ 75				
Men		0	≥ 90				
SSB/ fruit juices (servings/day)[d]	Sweetened soft drinks, energy drinks, fizzy drinks, diluted syrup, ice tea, alcoholic drinks substitutes, fruit juices, vegetable juices, smoothies	≥ 1	0	5.0 [0.6 – 10.0]	5.0 [0.0 – 10.0]	5.0 [0.6 – 10.0]	9.7 [4.8 – 10.0]
Nuts and legumes (servings/day)	Nuts, seeds, legumes, meat substitutes, soy products	0	≥ 1	0.0 [0.0 – 4.4]	0.0 [0.0 – 4.8]	0.0 [0.0 – 3.7]	0.0 [0.0 – 3.8]
Red/processed meat (servings/day)[d]	Fresh meat of mammals, offal, wild meat, sausages, cold cuts, smoked and cured meat	≥ 1.5	0	5.0 [2.8 – 8.7]	5.0 [2.4 – 8.6]	5.0 [3.2 – 8.7]	5.6 [3.7 – 9.1]
Trans fat (% of energy)[d]	Margarine, coco fat	≥ 4	≤ 0.5	10.0 [10.0 – 10.0]	10.0 [10.0 – 10.0]	10.0 [10.0 – 10.0]	10.0 [10.0 – 10.0]
Fish (g/day)	Fish, processed fish (fish in crumbs, ...), seafood, processed seafood (surimi, ...)	0	≥ 32.4	0.0 [0.0 – 5.0]	0.0 [0.0 – 5.0]	0.0 [0.0 – 5.0]	0.0 [0.0 – 5.0]
PUFA (% of energy)		≤ 2	≥ 10	2.9 [1.8 – 4.1]	2.8 [1.8 – 4.1]	3.0 [2.1 – 4.2]	2.7 [1.7 – 4.0]
Sodium (mg/day)[d]				4.8 [2.5 – 7.0]	4.6 [2.5 – 6.7]	5.3 [2.9 – 7.3]	5.1 [3.0 – 7.4]
Women		≥ 3337	≤ 1112				
Men		≥ 5271	≤ 1612				
Alcohol (drinks/day)[d]	Beer, wine, wine products, port, sherry, vermouth, cocktails, liquors, spirits			2.5 [2.5 – 6.2]	2.5 [2.5 – 6.2]	2.5 [2.5 – 5.0]	3.7 [2.5 – 6.2]
Women		≥ 2.5	0.5 - 1.5				
Men		≥ 3.5	0.5 - 2.0				

[a]Adapted from Chiuve et al. 2012 [1] and Chatelan et al. 2017 [2]

[b]Intermediate food intake was scored proportionately between the minimum and the maximum score

[c]Total score ranges from 0 to 110 points, with 0 indicating minimal adherence and 110 indicating maximal adherence

[d]For the components sugar-sweetened beverages/fruit juices, red/processed meat, trans fat and sodium, a high score corresponds to a low consumption; for the component alcohol, a high score corresponds to a moderate consumption

IQR: interquartile range; PUFA: polyunsaturated fatty acids; SSB: sugar-sweetened beverages

Table S2: Components of the Mediterranean Diet Score[a,b]

Component	Included food items	Criteria for minimum score (0)[c]	Criteria for maximum score (1)[c]	Overall Median [IQR]	German-speaking region Median [IQR]	French-speaking region Median [IQR]	Italian-speaking region Median [IQR]
Vegetables	All vegetables, leafy vegetables, sprouts, green beans, peas, sweat corn, root vegetables, cabbages, mushrooms, onions, seaweeds; Except: olives, herbs, vegetables juices	Below the median	Above the median	0.5 [0.0 - 1.0]	0.5 [0.0 - 1.0]	0.5 [0.0 - 1.0]	0.5 [0.0 - 1.0]
Legumes	Legumes	Below the median	Above the median	0.0 [0.0 – 0.0]	0.0 [0.0 – 0.0]	0.0 [0.0 – 0.0]	0.0 [0.0 – 0.0]
Fruits and nuts	All fruits, nuts, seeds; Except: fruit juices, fruit jams, candied fruit	Below the median	Above the median	0.5 [0.0 – 1.0]	0.5 [0.0 – 1.0]	0.5 [0.0 – 1.0]	0.5 [0.0 – 1.0]
Cereal	All bread products, flours, cereal flakes and bran, dough, pasta, rice, spätzle, other cereal grains (quinoa, barley, ...), cakes, biscuits, pies, potatoes, potato-based products (gnocchi, rösti, ...)	Below the median	Above the median	0.5 [0.0 – 1.0]	0.5 [0.0 – 1.0]	0.5 [0.0 – 1.0]	0.5 [0.0 – 0.5]
Fish	Fish, processed fish (fish in crumbs, ...), seafood, processed seafood (surimi, ...)	Below the median	Above the median	0.0 [0.0 – 0.5]	0.0 [0.0 – 0.5]	0.0 [0.0 – 0.5]	0.0 [0.0 – 0.5]
Meat	Fresh meat of mammals, fresh meat of poultry, offal, wild meat, sausages, cold cuts, smoked and cured meat	Above the median	Below the median	0.5 [0.0 – 1.0]	0.5 [0.0 – 1.0]	0.5 [0.0 – 1.0]	0.5 [0.0 – 1.0]
Dairy products	Milk, fermented milk drinks, buttermilk, yogurt, yogurt drinks, fermented milk and kefir, fresh cheese, soft cheese, hard cheese, cream, desserts made with dairy products, ice creams, sorbets	Above the median	Below the median	0.5 [0.0 – 1.0]	0.5 [0.0 – 1.0]	0.5 [0.4 – 1.0]	0.5 [0.5 – 1.0]
Alcohol				0.0 [0.0 – 0.5]	0.0 [0.0 – 0.5]	0.0 [0.0 – 0.5]	0.3 [0.0 – 0.5]

Women		< 5 g/day or > 25 g/day	5 - 25 g/day				
Men		< 10 g/day or > 50 g/day	10 - 50 g/day				
Fat intake	Ratio of monounsaturated to saturated fatty acids	Below the median	Above the median	0.5 [0.0 – 1.0]	0.5 [0.0 – 1.0]	0.5 [0.0 – 1.0]	0.5 [0.5 1.0]

[a]Adapted from Chatelan et al. 2017 [2]

[b]Total score ranges from 0 to 9 points, with 0 indicating minimal adherence and 9 indicating maximal adherence to the traditional Mediterranean diet

[c]Medians are sex-specific

IQR: interquartile range

Table S3: Weighted median [IQR] of each Alternate Healthy Eating Index (AHEI) component and differences between language regions (n=2,057)[a,b]

	**German-speaking region[e]**	**French-speaking region[e]**	**Italian-speaking region[e]**	**Overall**	**German vs. French**	**French vs. Italian**	**German vs. Italian**
	Median [IQR]	Median [IQR]	Median [IQR]	p-value[c]	p-value[c,d]	p-value[c,d]	p-value[c,d]
Vegetables	2.99 [1.62 - 4.51]	3.12 [1.71 - 4.78]	3.02 [1.46 - 5.17]	0.74	1	1	1
Fruit	2.69 [0.88 - 5.15]	3.00 [0.99 - 5.04]	2.79 [0.22 - 4.74]	0.37	1	0.50	0.80
Whole grains	2.98 [0.00 - 5.00]	2.48 [0.00 - 5.00]	0.00 [0.00 - 5.00]	**<0.01**	0.25	**0.03**	**<0.01**
SSB/fruite juces[f]	5.00 [0.00 - 10.00]	5.00 [0.59 - 10.00]	9.68 [4.85 - 10.00]	**<0.01**	0.15	**0.01**	**<0.01**
Nuts and legumes	0.00 [0.00 - 4.84]	0.00 [0.00 - 3.69]	0.00 [0.00 - 3.77]	0.09	0.33	1	0.20
Red/processed meat[f]	5.00 [2.37 - 8.61]	5.00 [3.19 - 8.75]	5.56 [3.75 - 9.11]	0.20	0.62	1	0.35
Trans fat[f,g]	10.00 [10.00 - 10.00]	10.00 [10.00 - 10.00]	10.00 [10.00 - 10.00]	**<0.01**	**<0.01**	0.35	**<0.01**
Fish[g]	0.00 [0.00 - 5.00]	0.00 [0.00 - 5.00]	0.00 [0.00 - 5.00]	**<0.01**	**<0.01**	1	**<0.01**
PUFA	2.85 [1.75 - 4.12]	2.99 [2.06 - 4.20]	2.66 [1.67 - 4.03]	0.17	0.80	0.19	0.65
Sodium[f]	4.58 [2.47 - 6.73]	5.29 [2.92 - 7.29]	5.14 [2.97 - 7.44]	**0.01**	**0.01**	1	0.23
Alcohol[f]	2.50 [2.50 - 6.25]	2.50 [2.50 - 5.00]	3.66 [2.50 - 6.25]	**<0.01**	**0.03**	**<0.01**	**0.01**

[a]The weighted median [IQR] of the participants is represented for each language region

[b]All results were weighted for sex, age, marital status, major area of Switzerland, nationality, household size, season and weekday

[c]p-values were derived from Kruskal-Wallis tests; bolded values represent statistically significant results (p-value<0.05)
[d]Bonferroni correction was applied to adjust for multiple testing
[e]German-speaking region: canton Aargau, Basel-Land, Basel-Stadt, Bern, Lucerne, St. Gallen, Zurich; French-speaking regions: canton Geneva, Jura, Neuchatel, Vaud; Italian-speaking regions: canton Ticino
[f]For the components sugar-sweetened beverages/fruit juices, red/processed meat, trans fat and sodium, a high score corresponds to a low consumption; for the component alcohol, a high score corresponds to a moderate consumption
[g]Similar values are due to a very skewed distribution
IQR: interquartile range; PUFA: polyunsaturated fatty acids; SSB: sugar-sweetened beverages

Supplementary references:

1. Chiuve, S. E.; Fung, T. T.; Rimm, E. B.; Hu, F. B.; McCullough, M. L.; Wang, M.; Stampfer, M. J.; Willett, W. C. Alternative dietary indices both strongly predict risk of chronic disease. *J. Nutr.* **2012**, *142*, 1009–1018, doi:10.3945/jn.111.157222.
2. Chatelan, A.; Beer-Borst, S.; Randriamiharisoa, A.; Pasquier, J.; Blanco, J. M.; Siegenthaler, S.; Paccaud, F.; Slimani, N.; Nicolas, G.; Camenzind-Frey, E.; Zuberbuehler, C. A.; Bochud, M. Major differences in diet across three linguistic regions of Switzerland: Results from the first national nutrition survey menuCH. *Nutrients* **2017**, *9*, 1–17, doi:10.3390/nu9111163.
